# Special Journals for Hospital Saturday Funds

**Published:** 1893-04-15

**Authors:** 


### SPECIAL JOURNALS FOR HOSPITAL SATURDAY FUNDS.

The Committee of the London Hospital Saturday Fund have commenced to publish a monthly journal on similar lines to *Forward*, the monthly journal of the Birmingham Hospital Saturday Fund. The experience of the latter journal, which has just completed its first year, may therefore prove of interest. We quote their own words: "It has signally failed in several respects. First, it has not proved a channel for the expression of opinions from the workshops on matters connected either with the Hospital Saturday Fund or the institutions which that Fund so largely supports. In the next place, it has failed to be a medium for the communication of information from the institutions themselves to the workshops. Although each institution was asked to furnish any intelligence as to its affairs which would be likely to foster interest amongst the subscribers, not a single response has been received. This, too, is to be regretted. The Medical Institutions have come to regard the Hospital Saturday contribution as a matter of course. The delegates whom they appoint on the Board enter into the spirit of the movement so little that they seldom attend the meetings; and the attitude of the committees, or at any rate of the officials of certain of them, can in some cases hardly be described as other than hostile. It is unfortunate that such should be the case. Success invariably excites jealousy, and it is probable that the unparalleled position which the movement has attained is the cause of the unfriendly position taken up towards it in quarters where it should receive cordial sympathy and support. Not infrequently may the remark be heard that the Hospital Saturday Fund is getting too powerful. So long as its power and influence are exercised wisely, it is difficult to see with what arguments the contention for their limitation can be supported."

The editor of *Forward* writes: "The unanimous feeling among the members of the Executive Committee is that *Forward* is a most valuable aid to the work of the Association. If the delegates express a similar opinion, the publication of the journal will be continued during the ensuing year, and will be issued on the first of each month, instead of the sixteenth as heretofore. The latter date was fixed in order that reports of the meetings of the Home Club and Executive Committee, which are respectively held on the first Monday and second Tuesday in the month, might appear. It is, however, considered that its issue at the beginning of the month will improve its prospects, hence the alteration. It is only necessary that we should add an appeal to all interested in the Hospital Saturday Fund that, if its publication be continued, they shall give the journal their constant support—not only by becoming subscribers, but by forwarding communications for publication upon any matter which they consider deserves attention. We want *Forward* to be the organ of the artizan classes, and to give expression to their opinions."

The first number of the Metropolitan "Hospital Saturday Fund Journal" has appeared. If imitation be the sincerest flattery, then the Birmingham authorities have cause for congratulation. We venture to think that if it is desirable to publish such a journal it should be of sufficient size to enable the editor to publish matter of general interest with each number. Four quarto pages of which more than one is devoted to advertisements, published once a month, cannot under any circumstances be made to contain what such a journal should contain, if it is to be of any practical help to the Council of the Metropolitan Hospital Saturday Fund. We venture, therefore, to hope, that those who are responsible for this publication will at once take steps to increase the size of the journal to at least eight pages, and that every number will in future contain at least one good article dealing with a subject calculated to arouse the attention of the reader, whilst it will serve to educate in sound principles of hospital administration, the thousands of subscribers to the fund to which the editor alludes in the first number. We are sure courage will prove of the essence of success, and whilst welcoming the "Hospital Saturday Fund Journal" we desire to give it form and substance by emphasising the recommendations we have ventured to make in regard to its conduct and size. We would further point out that steps should be taken to circulate the journal by sending it to representative newspapers and individuals, as this point seems to have been so far overlooked or delayed until it was too late to render the material service it might otherwise have done.

